# Hydrogels for Salivary Gland Tissue Engineering

**DOI:** 10.3390/gels8110730

**Published:** 2022-11-10

**Authors:** Sangeeth Pillai, Jose G. Munguia-Lopez, Simon D. Tran

**Affiliations:** McGill Craniofacial Tissue Engineering and Stem Cells Laboratory, Faculty of Dental Medicine and Oral Health Sciences, McGill University, 3640 Rue University, Montreal, QC H3A 0C7, Canada

**Keywords:** salivary glands, hydrogels, ECM, mechanotransduction, signaling pathways

## Abstract

Mimicking the complex architecture of salivary glands (SGs) outside their native niche is challenging due their multicellular and highly branched organization. However, significant progress has been made to recapitulate the gland structure and function using several in vitro and ex vivo models. Hydrogels are polymers with the potential to retain a large volume of water inside their three-dimensional structure, thus simulating extracellular matrix properties that are essential for the cell and tissue integrity. Hydrogel-based culture of SG cells has seen a tremendous success in terms of developing platforms for cell expansion, building an artificial gland, and for use in transplantation to rescue loss of SG function. Both natural and synthetic hydrogels have been used widely in SG tissue engineering applications owing to their properties that support the proliferation, reorganization, and polarization of SG epithelial cells. While recent improvements in hydrogel properties are essential to establish more sophisticated models, the emphasis should still be made towards supporting factors such as mechanotransduction and associated signaling cues. In this concise review, we discuss considerations of an ideal hydrogel-based biomaterial for SG engineering and their associated signaling pathways. We also discuss the current advances made in natural and synthetic hydrogels for SG tissue engineering applications.

## 1. Introduction

**Figure 1:**
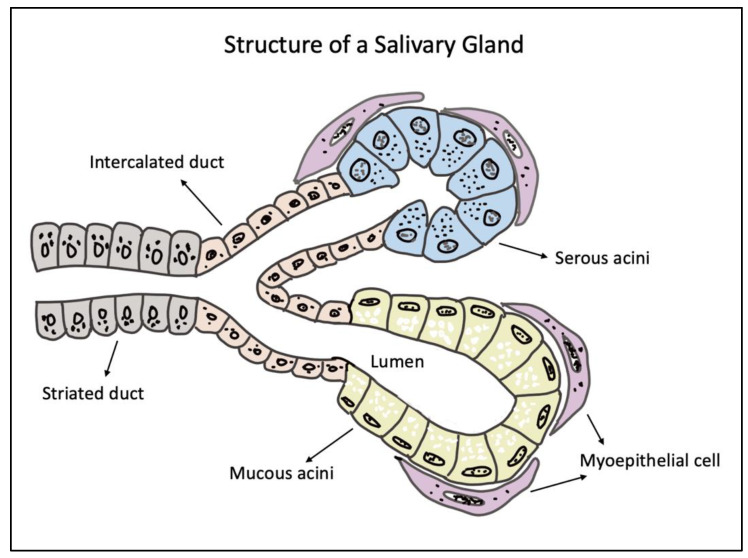
Schematic diagram of a salivary gland structure showing the secretory end pieces made of mucous and serous acini, supported by overlying myoepithelial cells. The secretory units extend to form the intercalated, striated, and excretory ducts through which saliva passes into the oral cavity.

Salivary glands (SGs) are exocrine organs which are responsible for the production and secretion of saliva—a complex, multifunctional, extracellular fluid that helps in the maintenance of oral homeostasis. In humans, SGs are categorized into major and minor glands, which are mainly based on the size of the gland and the volume of saliva they secrete [1]. There are three pairs of major SGs, namely, the parotid (PG), the submandibular (SMG), and the sublingual glands (SLG) [2,3]. The anatomical architecture of all three major SGs is essentially the same, with an arborized ductal system that opens into the oral cavity comprised of secretory end pieces, called the acini, responsible for producing saliva [4]. These three major SGs account for more than 90% of the salivary secretions, and the remaining 10% is complemented by the hundreds of minor SGs present in the upper digestive tract mucosa, such as the lower lip, palate, tongue, cheeks, etc. [5]. Among the major SGs, the SMG and SLG contribute to nearly 80% of ‘unstimulated saliva’ production. The PG being the largest major SG is only responsible for 20% of the unstimulated secretions [6]. Structurally, the SGs consist of parenchymal cells (acinar, ductal, and myoepithelial cells) and the stroma (supporting connective tissues) [3,7]. Depending on the type of major SGs, the acinar cells can be serous or mucous in nature. The PG has serous acini, whereas the SLG and minor SGs consist of mucous type acini, with the SMG comprising both serous and mucous acini. The acinar cells are called the secretory cells in the SGs, as they are chiefly responsible for saliva production. The ductal cells, on the other hand, can be cuboidal cells which form the intercalated ducts or columnar cells with striations forming the striated ducts which finally extend to form the excretory ducts [8]. The myoepithelial cells lie between the basal cell membrane of the acinar and the striated and intercalated ductal cells. They play a crucial role in contracting the acinar cells in response to parasympathetic and sympathetic stimulation to secrete saliva [9]. Apart from these adult epithelial cells, the SG also hosts stem/progenitor cells that are responsible for the self-duplication, renewal, and differentiation into adult cells in response to injury or stress [10]. The stromal component of the SGs is mainly comprised of fibroblasts, collagens, the neurovascular bundle, and the adipocytes [8]. In addition, the non-parenchymal elements are supported by immune cells, such as T-cells, macrophages, monocytes, and lymphocytes, which maintain the overall health of the native SG [8]. Figure 1 depicts a schematic illustration of a normal SG structure indicating the major cell types.

Mimicking the complex architecture of the SGs outside its native niche is quite perplexing. Over the past two decades, significant progress has been made to recapitulate the gland structure and function using several in vitro and ex vivo models [11,12,13,14,15,16,17]. Initial studies aimed at engineering an artificial SG used simple tissue culture plates, which was followed by the use of protein-coated substrates to grow salivary epithelial cells [18,19]. These studies used polymeric substrates such as Poly-L-lactic acid (PLLA), Polyglycolic acid (PGA), Poly lactic-co-glycolic acid (PLGA), either alone or in combination with extracellular matrix (ECM) proteins such as fibronectin, laminins, and collagens, to grow SG cells [20]. Further, the use of transwell filters allowed a compartmentalized culture of polarized SG epithelial cells with the expression of key functional proteins and fluid movement [11]. However, due to the inaccurate representation of the complex SG structure and their limited functionality in two-dimensional (2D) cultures, these approaches were succeeded by use of natural and synthetic matrices and scaffolds to grow SG cells in three dimensional (3D) cultures. Among these available strategies, hydrogel-based culture of SG cells has seen a tremendous success in terms of developing platforms for cell expansion, building an artificial gland, and for use in transplantation to rescue loss of SG function. Hydrogels are polymers with the potential to retain high volumes of water inside of their three-dimensional structure, thus responding to external stimuli by closely simulating the architecture and composition of the ECM. The ECM properties are essential for the cell and tissue integrity and for the maintenance of complex interactions within an organ system [21]. These factors can enhance the hydrogel properties in supporting a fully functional gland.

In this narrative review, we discuss the criteria for tuning an ideal hydrogel-based biomaterial for SG tissue engineering based on our current knowledge of other highly branched organs systems. We mainly discuss the different hydrogel properties, such as their biocompatibility, stiffness, pore size, architecture, etc., that suit specific SG cell functions and their association with key mechanotransduction proteins and pathways. Finally, we oversee the current advances made in natural and synthetic hydrogels including our contributions to SG tissue engineering applications.

## 2. Tuning Ideal Hydrogel Properties for SG Tissue Engineering

**Figure 2:**
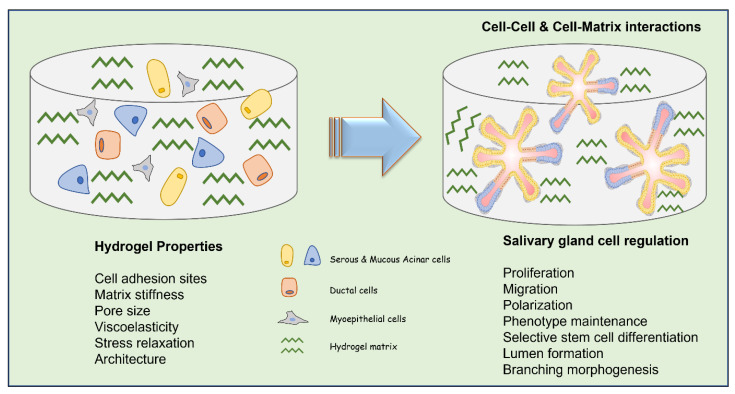
Schematic illustration of different types of SG cells within a hydrogel matrix describing the key hydrogel properties to be considered to adequately regulate cell functions for SG tissue engineering applications.

In general, an ideal biomaterial used in SG tissue engineering should: (1) support the cell proliferation and migration within the matrix, (2) maintain the phenotypic characteristics of SG cells, (3) stimulate selective differentiation of SG stem/progenitor cells, (4) enable cell assembly and reorganization (cell polarization and lumen formation), (5) support matrix remodeling (permissible hydrogels), and (6) allow duct expansion (promote branching morphogenesis) (Figure 2). For bioengineering an artificial SG, the biomaterial strategy depends on the type of cells used such as cell lines [22], primary single cells [11,23], cell clusters such as salivary functional units [14], acinar cell clusters and intercalated ducts [15], the tissue derivation (adult or embryonic- epithelium or mesenchyme) [24], and the expected functions of the model. The three main types of SG cells include the acinar, ductal, and myoepithelial cells and, so far, there is no fully characterized description of an ideal biomaterial that can foster all adult epithelial cells simultaneously to fulfil their function or to promote the selective differentiation of available stem/progenitor cells and their organization within a matrix. Most research in this field focusses on the use of either of these cells (mainly primary human or rodent derived) and or stem/progenitor cells with a bioactive scaffold to generate an SG mimetic [17,25,26].

### 2.1. Hydrogel Biocompatibility

To successfully fulfil the abovementioned criteria, we need to consider two crucial parameters in terms of biomaterial selection: (1) the biochemical/bioactive component and (2) the mechanical-physicochemical properties of the hydrogels. The hydrogels must be biocompatible, stable, and biodegradable and support living cells, biological factors (such as growth factors), and ensure the correct nutrient and oxygen exchange. They should be developed using mild preparation strategy and note should be taken that the hydrogel degradation by-products should not be toxic to cells or microtissues [27].

### 2.2. Presence of Cell Binding Motifs

The presence of anchorage sites or specific cellular binding motifs within the hydrogel matrix/scaffold are pivotal to support adhesion proteins or junction proteins to ensure the establishment of the subsequent steps in cell biology processes such as proliferation, migration, self-assembling, and, most importantly, polarization. The absence of adhesion sites driving to poor/null cell attachment can potentially lead to anoikis of the cells [28]. SG epithelial cells are characterized by their ability to form cell–cell adherent junctions using cadherins and β-catenins. These proteins reinforces the cell cytoskeleton via actin filaments [29], and, further, they form tight junctions apico-laterally by means of protein complexes such as occludins and claudins. Together, these junctions promote the epithelial cell integrity, apico-basal polarity, and maintenance of ion transport and fluid movement [29]. ECM-derived hydrogels contain these motifs, such as RGD (Arginine-Glycine-Aspartic Acid), DGEA (Aspartic acid-Glycine-Glutamic acid-Alanine), YIGSR (Tyrosine-Isoleucine-Glycine-Serine-Arginine) [30], and heparan sulfate binding domains, which inherently support cell attachment, as opposed to natural bioinert materials such as alginate. So far, limited studies have evaluated the specific benefits of modifying and integrating binding ligands for SG three-dimensional culture [31,32].

### 2.3. Hydrogel Stiffness

Appropriate hydrogel stiffness is a key aspect which regulates all cell activities and functions including cell phenotype maintenance [33], proliferation [34], differentiation [35], and stemness [36]. The cell–ECM interactions are regulated based on the substrate/ECM stiffness, which provides instructions to the mechanosensors, such as focal adhesions kinases (FAKs), integrins, stress fibers, etc. [37,38]. In SG modelling, this property of hydrogel plays a key role in the cytoskeleton contractility of the cells. The cytoskeletal properties are variable in different SG cell types and can be evaluated by monitoring actin stress fibers, integrins, and focal adhesion (FA) complexes. The cytoskeleton response to stiffness will be different for a pyramidal acinar cell than a tall columnar ductal cell or a stellate-shaped myoepithelial cell. These cell mechanosensors take part in a cascade of signaling pathways in response to varying stiffness, which eventually dictates the cell fate and function. In SG ex vivo cultures, especially for embryogenic tissues, it has been observed that softer gels/substrates promote phenotypic retention and differentiation of the cells [24].

A variety of signaling cascades are activated in response to mechanical stimuli from the ECM, of which the most important pathways that might determine the hydrogel characteristics for SG engineering include the FAK, Rho Kinase (ROCK), Yes Associated Protein (YAP), and Wnt/β-catenin signaling. Sequeira et al. isolated and cultured embryonic SMGs on PLGA nanofibers and evaluated branching morphogenesis, epithelial proliferation, and FA protein expression [39]. They found extensive budding and ductal outgrowth from the organ explants, with strong expressions of FAKs, talin, p-paxillin, and vinculin in the basal membranes. However, the expression of these FA proteins was low for adult fully differentiated acinar cells growing on PLGA nanofiber scaffolds [39]. It is well established that ROCK signaling is essential for SG morphogenesis during development [40], and it also plays a critical role in delaying senescence and promoting epithelial cell proliferation in SG cultures [41]. Supporting this evidence, Koslow et al. tested the effect of ROCK inhibitors on adult SG organoid cultures. The authors found an increment in proacinar markers such as Kit and Mist-1 and the maintenance of the SG progenitor population [42]. Several other groups have shown that higher ECM stiffness leads to cell spreading and higher FA generation in progenitor cells [43], causing the accumulation and clustering of integrins. The overexpression of FA proteins drives a loss in basal polarity and challenges in lumen formation and acinar reorganization in three-dimensional SG cultures. This phenomenon is analogous to mammary gland tumors, where tumor progression occurs due to the activation of FAK and ROCK pathways as a consequence of the increased cell generated forces in response to high stiffness in the tumor microenvironment [44]. However, while these robust cell–ECM interactions might affect cell–cell interactions supported by adherent and tight junctions, it is important to note that recent results from single cell RNA sequencing on embryonic SG cells suggest the need for a higher integrin mediated interaction, rather than only E–cadherin junctions. The weak cell–cell and strong cell–ECM interactions are essential for inducing surface expansion and branching morphogenesis in SG three-dimensional cultures [45].

YAP is a key protein involved in tissue regeneration in various organs [46]. Ectopic YAP expression has been shown to induce progenitor/stem cell expansion and dedifferentiation of adult epithelial cells in branched organs such as mammary glands and the pancreas [47]. YAP activation plays a crucial role in SG development. The deletion of the YAP gene in mice resulted in a lack of epithelial patterning in the developing gland [48]. Rocchi et al. reported that YAP overexpression resulted in the stem/progenitor cell mediated SG organoid expansion in three-dimensional cultures [49]. They also reported that translocation of YAP to the nucleus can induce activation of new ductal cells, which can be utilized for SG regeneration after severe radiation injuries [49]. It is noted by other researchers that this cytoplasm-to-nuclear translocation of YAP occurs in response to an increment in the ECM stiffness, leading to the proliferation of different cell types such as fibroblasts [50].

Similarly, the Wnt/β-catenin signaling is a well-studied pathway in SG biology and is known to play a crucial role in SG stem cell maintenance and long term expansion in vitro [51]. In this regard, it has been shown that β-catenin translocation to cell nuclei is promoted in response to higher matrix stiffness [52]. While each of these mechanotransduction pathways are involved in different SG development roles/steps, it should be noted that stiffness-associated cell functions and their signaling cascades on a hydrogel-coated surface (such as 2.5D/on top cultures) are different from three-dimensional ECM hydrogels. To successfully utilize these mechanical stimuli and convert them into biological signals, it is essential to promote selective stiffness, depending on cell types (fully differentiated adult epithelial cells versus proacinar/stem/progenitor cells) and the platform used.

### 2.4. Hydrogel Pore Size

The ability to fabricate hydrogels with varying pore sizes constitutes another reason for their use as cell scaffolds and three-dimensional culture platforms. Three-dimensional matrix pore size is determinant in multicellular organization, migration, and transport of nutrients and soluble cues within its three-dimensional architecture [53]. This is especially important in the SG organ system where the cells vary in shape and size. Having a uniform pore size might limit the cell migration and reorganization inside a three-dimensional structure, driving the activation or inactivation of crucial signaling pathways that play a role in SG development. It has been reported that an increment in the matrix pore size influence the cell membrane protein based force transmission and cytoskeleton rearrangement [54]. This instigates the need to develop hydrogels with more permissible matrices allowing ECM remodeling. In this context, Ozdemir et al. described the use of thiolated and acrylated hyaluronic acid (HA) based hydrogels to create a molecular weight mismatch and thus a covalently crosslinked permissible HA matrix to support multicellular spheroid formation of SG stem/progenitor cells [55].

Generally, smaller hydrogel pore sizes have been shown to cause diffusion of FA complexes and proteins into cell cytoplasm, as opposed to into the cell membrane [56]. A similar behavior is seen with the translocation of mechanosensing protein YAP within the cell cytoplasm. Spatial confinement due to small pore sizes can lead to YAP retention in the cell cytoplasm, which can potentially affect the SG stem/progenitor cell expansion, recruitment, and can inhibit the dedifferentiation of adult epithelial cells [49,57]. The nuclear morphology is different among various SG cell types. It is well established that cell cytoskeleton and nuclear morphology are closely regulated by ECM forces based on both their stiffness and geometry [58]. The ECM forces acting on the cell cytoskeleton is further transmitted to the nucleus of the cell, thus remodeling the nucleoskeleton, which affects the cell chromosome and chromatin dynamics [59]. Understanding these dynamics is essential for SG cell migration within the hydrogels, as the nuclear size often becomes the limiting factor. Makhija et al. evaluated the changes in nuclear deformability and telomere dynamics by altering the cell cytoskeleton organization using a fibroblast cell line. The results revealed that the nuclei of the cells with a constrained isotropic geometry are more deformable than cells with an elongated and polarized geometry [60]. These results point towards important considerations in defining how polarizable SG epithelial cells migrate within the hydrogel matrix. For example, elongated columnar ductal cells might not be able to squeeze through the hydrogel matrix pores and migrate at the rate similar to the smaller cuboidal cells, whereas myoepithelial or progenitor cells with shorter and rounded cell skeletons can. This challenge is also supported by the observation that adult SG primary epithelial cells, when dispersed as single cells, fail to migrate and form acinar-like clusters if the hydrogels are densely crosslinked [61]. In a nutshell, hydrogel pore size plays a major role in determining not only the specialized cell fate, but the genomic stability due to cytoskeleton induced nuclear deformations, which can affect cell functions such as DNA damage response and telomere function [62]. These factors should be kept in mind while designing hydrogel networks for SG three-dimensional culture to study diseases such as radiation response and for drug screening applications.

### 2.5. Viscoelasticity and Hydrogel Architecture

Tunable viscoelastic properties are necessary to support and harbor the cells to promote spreading, proliferation, and differentiation [63,64]. The stress relaxation in these hydrogels is needed to support cell-induced matrix remodeling, which is a decisive attribute in cells reorganizing into SG acinar-like clusters in three-dimensional matrices. Stress relaxation is also fundamental for the accumulation of cell binding sites and, thereby, recruitment of β-integrins, FA formation, and further YAP localization [64,65].

**Figure 3:**
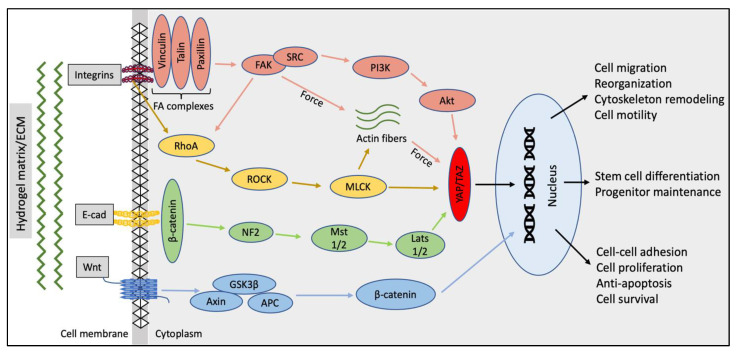
Schematic map of the key signaling pathways to be considered while designing hydrogel properties (binding sites, stiffness, pore size, architecture, etc.) for SG tissue engineering and their association with the cell fate and function [70,71,72,73,74].

Hydrogel architecture plays an essential role in defining how the cell self-assembles into a regulated functional, complex three-dimensional structures in cultures [66]. SG epithelial cells vary their phenotypic characteristics in response to different physicochemical stimuli, such as matrix-mediated cell forces, supplements (such as serum), or growth factors. It has been observed that cells growing on aligned or microfibrous scaffolds have a tendency to spindle up, as opposed to the ones grown on nanofibers or non-aligned matrices where cells attain a more rounded phenotype [66]. In addition, the non-aligned fibers also seem to promote a faster cell migration than the former [67]. The aligned fibers (microfibers 1–4 um) seem to create a massive FA complex formation and subsequent FAK activation [68]. These findings have been previously confirmed by Sequeira et al., wherein they observed that nanofibrous PLGA (250 nm) scaffolds, unlike microfibrous (1200 nm) PLGA, lead to decreased FAK activation and talin expression in acinar and ductal SG epithelial cells. These results were concurrent with the native adult SG physiology and cellular reorganization pattern [39]. The same group further advanced the use of PLGA nanofibers to coat micropatterned craters on poly dimethyl siloxane wafers to utilize their low FA complex formation, leading to cell reorganization with better apico-basal polarization and tight junction formation [69]. The results indicate the nature of SG epithelial cells to migrate faster and reorganize into acinar cell clusters as a result of less FA complexes formed with nanofiber scaffolds. As opposed to this, in microfibrous scaffolds, the architecture of the matrix may cause higher transmission force to the SG cells. These forces could lead to formation of a larger actin network, preventing the cells from easily migrating within the scaffolds. The limited cell movement can further lead to an increase in the local FA production, thereby strengthening cell–ECM adhesion, thus limiting cell reorganization. In general, hydrogel fiber dimensions, along with other parameters and their signaling pathways, can play a significant role in SG cell modulation, migration, and reorganization (Figure 3). The hydrogels/ECM should be designed by keeping in mind the expected application of these hydrogel-derived SG structures and their end point evaluation strategies [70].

## 3. Advances in Hydrogel Systems for Building an Artificial SG

Several biomaterials have been used to culture mammalian cells for tissue engineering and regenerative applications. There are more comprehensive reviews about different types of hydrogels and their applications in tissue engineering using different cell types, such as breast cancer cells, neuronal cells, pancreatic tissue, etc. [21,30,75,76,77]. However, hydrogels for bioengineering artificial SG models are still under development, limiting the information regarding the usage of biomaterials as three-dimensional scaffolds for SG cells. In this section, we will outline recent advances in natural and synthetic hydrogel-based platforms for culturing SG cells.

### 3.1. Naturally Derived Hydrogels

Matrigel is a commonly used biomaterial in 3D culture. In the SG field, Matrigel plays a critical role in SG development and morphogenesis [78], promoting primary SG cell attachment and differentiation in either 2.5D or three-dimensional culture [12,79]. Studies show that Matrigel promotes the assembly of acinar-like structures in 3D culture while expressing key SG proteins such as AQP-5 and amylase and maintains tight junction-associated proteins specific to SG epithelial cells. The low stiffness of Matrigel, when used in lower concentrations (e.g., 2 mg/mL), drives the cell differentiation and phenotypic maintenance. Even when Matrigel has been used widely in 3D culture, it represents several limitations for tissue engineering applications, such as poor mechanical properties and variability batch-to-batch leading to differences in mechanical attributes and biological composition [80]. Additionally, Matrigel, for being an animal derivative material, cannot be used for clinical translation due to negative immunological response from the host. Also, the numerous basement membrane (BM) proteins present in Matrigel make it difficult to understand the specific effects of each component in regulating SG cell behavior.

Other BM-derived peptides, such as laminin in combination with fibrin hydrogels, have shown to promote SG spheroid formation, branching, and functionality [81]. Fibrin gels are made by combining fibrinogen and thrombin at 37 °C, thus creating mechanically tunable gels by modifying the ratio of these components. On the other hand, laminin is generally incorporated into synthetic materials to improve cell attachment [82]. Laminin peptides chemically conjugated with fibrin gels allow the formation of acinar-like clusters showing polarization, lumen formation, and calcium signaling. The studies conducted in vivo using this system also show an increment in acinar differentiation markers and the restoration of the salivary flow rate in mice [83]. However, it has been found that certain sequences in the laminin-1 peptide can lead to tumorigenesis and immunogenic responses, thus hindering its use in clinical applications.

Other naturally occurring protein-derived hydrogels, such as collagen, fibrin gel, or their combination, have been used to culture SG cells to form acinar- or ductal-like structures [84,85]. Even if these materials support SG cell cluster formation and the expression of SG specific markers, maintaining a differentiated cell state and further promoting branching morphogenesis require additional growth factors. The lack of tunability of collagen-based materials, in addition to their weak mechanical strength during cell traction, limit their use in long-term SG culture systems.

Chicken egg derivatives have been used to create hydrogels for SG culture as an accessible, low-cost alternative to current three-dimensional hydrogel-based platforms. Our lab has tested egg yolk plasma (EYP) and egg white material (EW) to grow immortalized human SG acinar cells (NS-SV-AC) and human SG fibroblasts inside of EYP gels. Cells mixed within EYP showed high viability, while the NS-SV-AC reorganized as a spheroid-like structure while fibroblasts formed sheet-like structures within the EYP gel. Mixing both EYP and EW also promoted the proliferation of three-dimensional SG spheroids. However, using EYP as a gel for SG culture is not ideal for imaging and spheroid tracking due to the opacity of the gel [86,87]. Further studies were performed using EW and alginate as composites to grow SG cells in 2.5D platforms. By modifying the concentration of alginate in the final blend, it is possible to control the size of the SG spheroids formed, which show high viability and proliferation and exhibit spheroid sizes comparable to the ones growing in Matrigel [88]. These results position the egg-derived hydrogels as an affordable, reproducible SG platform for future tissue engineering applications.

Gelatin, a product derived from the partial hydrolysis of collagen, is a suitable material for biomedical applications due to its biodegradability, biocompatibility, and reabsorption by the host. The viscoelastic property of this polymer allows cell-mediated stromal remodeling (such as in SG acinar assembly) by deposition of ECM proteins. The limited temperature window for its sol–gel transition makes its manipulation difficult and limits its use in bioprinting when large, complex three-dimensional models are built up at room temperature. Combining gelatin with other polymers allows one to improve their mechanical properties, such as the use of crosslinkers such as genipin or the addition of methacrylate. Gelatin-methacrylamide (GelMA), with other combinations such as polycaprolactone (PCL), silver ink, or alginate, has been used widely in tissue engineering applications. Cells grown in GelMA composites have shown the ability to proliferate, maintain viability, differentiate, and perform key cell functions [30].

Alginate is another naturally occurring bioinert material used in several tissue engineering applications. This carbohydrate enables mechanical reinforcement that allows the development of gels with controlled stiffness via ionic crosslinking using divalent ions (such as calcium). This reaction is reversible by applying highly coordinated divalent ions chelators (such as citrates or EDTA), allowing the recovery of pure three-dimensional spheroids/organoids for future tissue engineering applications [89]. The crosslinking reaction also produces nanopore structures which mimic BM and thus have the potential to support cell spreading and migration [90]. Besides, the use of low molecular weight alginate, or variations in the final concentrations on the polymer, allows one to tune the viscoelastic properties and stress relaxation time, which makes an ideal material to induce cell contraction-based ECM remodeling in three-dimensional SG culture. Unfortunately, alginate lacks cell adhesion moieties and thus lacks crucial cell adhesion, attachment, and migration when used alone. This limits its application in promoting SG cell–cell interactions, which are mainly mediated by tight junction proteins [91]. To overcome this, alginate has been used either with chemical modifications to contain adherent peptide motif sequences (e.g., RGD) or in combination with other natural biomaterials such as gelatin and collagen [92,93]. These modifications provide cellular binding sites, which are necessary for cell–cell and cell–matrix adhesions.

HA is a hydrophilic non sulphated glycosaminoglycan (GAG) which is found in the ECM, especially surrounding the mesenchyme. HA is bioactive with several cell binding sites for different cell surface receptors which direct cell adhesion, migrations, and morphogenesis [94]. CD44, a cell surface glycoprotein, acts as a receptor for HA and allows for cell binding using these moieties. Our group has previously shown that the CD44 protein localizes to the serous acinar cells in normal human SGs tissues [95]. This principle was applied by another research group where they used HA-based hydrogels with an elastic modulus of 60–100 Pa to encapsulate human SG primary cells which showed self-assembly into acinar-like clusters. These clusters were determined to be functionally active by amylase activity and calcium stimulation [17]. However, the molecular weight (MW) of HA is an important factor in designing hydrogels due to the inconsistent CD44 clustering, and it has been shown that high MW HA can inhibit angiogenesis and inflammatory responses [96]. Several groups have utilized the highly permissible nature of chemically modified HA hydrogels to create SG mimetics in three dimensions [16,55,97,98].

### 3.2. Synthetic Hydrogels

While naturally derived hydrogels provide inherent biological properties, such as bioactivity, biocompatibility, biodegradability, and cell signaling properties leading to cell survival and performance, their batch-to-batch variability and the presence of various endogenous epitopes and cues within these biomaterials largely affect cell function. Synthetic biomaterials with an exact known composition with high tunability and reproducibility have been used for SG tissue engineering.

PLGA and PEG (polyethelene glycol) have been tested as synthetic biomaterials in SG engineering [39,61,99]. PLGA has been shown to support embryonic SG by promoting branching morphogenesis in dissected embryonic glandular tissue and in adult epithelial cells to express acinar-specific markers such as AQP-5. These are important characteristics in modelling an artificial SG. However, it has also been observed that the SG structures within these hydrogels become disorganized once they are highly confluent. While PLGA can be processed into any shape or size and encapsulate growth factors, its degradation products are acidic in nature and difficult to remove from the human body, thus limiting its use in the clinical transplantation of artificial SG [100].

Polymerized PEG, using methacrylate, has been shown to induce significant loss of submandibular gland cell viability. This challenge was overcome by thiol-ene polymerization, which led to better SG cell encapsulation. Still, both types of hydrogels failed to re-organize primary single cells into SG cell clusters or organized acini [61]. PEG has also been used in combination with HA to encapsulate and culture primary human SG cells. These cells were able to reorganize into acini-like structures and further the introduction of PlnDIV (domain IV of perlecan) peptide promoted lumen formation in these SG three-dimensional cultures [16]. Hydrophobic polymers such as PVDF (poly vinylidene fluoride) and silk fibroin used as porous scaffolds have also shown results supportive of cell growth and phenotype retention [101]. However, there is limited evidence of their use in long-term SG three-dimensional culture for clinical translation.

Polycaprolactone (PCL) is another synthetic material used as a scaffold in periodontal and bone tissue engineering due to its high mechanical strength [82]. A robust and functional acinar-like organoids three-dimensional culture system was established by using PEG-micropatterned PCL nanofibrous microwells. Human parotid epithelial cells grown on PEG/PCL microwells self-assembled as three-dimensional structures, expressing higher levels of SG epithelial markers (α-amylase and AQP5), tight junction proteins (ZO-1 and occludins), adherence junction protein (E-cadherin), and α-amylase secretion and intracellular calcium concentration compared to Matrigel [102]. However, the hydrolytic degradation of PCL under in vivo conditions is considerably slow, representing a disadvantage in future clinical applications where polymer degradation and elimination needs to be much quicker.

Other strategies employed in SG engineering are the use of well defined SG acinar spheroids derived from one of these above-mentioned hydrogels and their transfer into micro- or nano-fabricated templates. A seminal work using matrix metalloproteases (MMP) degradable PEG hydrogels with micro bubble (MB) array technology was recently reported [15]. This bubble architecture was shown to maintain the SG acinar niche and allowed long term culture of primary SG cells for use in high content screening. Although the platform supports functional characteristics of SG cells, it relies mainly on the acinus secretory unity with a few myoepithelial and ductal components. There is still a lack in addressing the multicellular SG milieu with appropriate ECM to support vascular and neural stimuli, in addition to soluble cues and growth factors for their maintenance.

## 4. Conclusions and Future Directions

To conclude, to successfully devise a hydrogel for the purpose of developing a fully functional multicellular SG, it is essential to consider several key factors. These include, but are not limited to, the hydrogel composition, preparation, availability of binding sites, stiffness, pore size, stress relaxation, and matrix architectural features. Most hydrogel-based matrix/scaffold development focuses on successfully creating functional SG organoids or spheroids by self-reorganization of epithelial cells or progenitor cells, exploiting low FA-mediated interactions with ECM and high cell–cell interactions forming adherent and tight junctions. However, recently published results from single-cell RNA sequencing of embryonic SGs shines evidence on the need for maintaining a low cell–cell interaction and robust cell–matrix integrin mediated signaling to promote budding and branching morphogenesis. Combined, these findings suggest that no one single hydrogel composition can attain all the canonical physicochemical interactions necessary to replicate each stage of SG morphogenesis. It is fundamental to independently study and understand the mechanical cues required by each SG cell type, following their subsequent interaction with the ECM microenvironment to regulate cell function and fate. Utilizing our current knowledge of cell modulation via mechanotransduction from other branched, multicellular organ systems, combined with advances in three-dimensional SG culture, their associated signaling pathways, and soluble cues, could potentially progress our ultimate goal of engineering a SG. Additionally, a combination of hydrogel platforms could be useful to first construct separately a secretory acinar and ductal branching units with their respective niche associated progenitor/stem cell markers. These structures can then be re-adapted by providing adequate mechanosignalling for each outcome desired, which can be combined using the recent advances in multicellular-multimaterial three-dimensional bioprinting and integrated chip systems to develop a fully functional SG.

## Abbreviations

2D	Two-dimensional
2.5D	Two-point-five-dimensional
3D	Three-dimensional
Akt	Protein kinase B (PKB)
APC	Adenomatous polyposis coli
AQP-5	Aquaporin 5
BM	Basement membrane proteins
CD44	Cluster of Differentiation 44
DGEA	Aspartic acid-glycine-glutamic acid-alanine
ECM	Extracellular matrix
EDTA	Ethylenediaminetetraacetic acid
EW	Egg White
EYP	Egg Yolk Plasma
FA	Focal adhesion
FAKs	Focal adhesion kinases
GelMA	Gelatin-methacrylamide
GSK3β	Glycogen synthase kinase 3 beta
HA	Hyaluronic acid
Kit	Tyrosine-protein kinase (CD117)
Lasts 1/2	Large tumor suppressor 1 and 2
Mist-1	Muscle, intestine and stomach expression 1
MLCK	Myosin light-chain kinase
Mst 1/2	Mammalian sterile 20-like kinase 1 and 2
MW	Molecular weight
NF2	Neurofibromatosis type 2
NS-SV-AC	Normal human SG-simian virus 40-acinar cells
PCL	Polycaprolactone
PEG	Polyethelene glycol
PG	Parotid gland
PGA	Polyglycolic acid
PI3K	Phosphatidylinositol-3-kinase
PLLA	Poly-l-lactic acid
PLGA	Poly-l-lactic-co-glycolic acid
RGD	Arginine-glycine-aspartic acid
RhoA	Transforming protein RhoA
ROCK	Rho kinases
SGs	Salivary glands
SLG	Sublingual gland
SMG	Submandibular gland
SRC	Proto-oncogene tyrosine-protein kinase
TAZ	Transcriptional co-activator with PDZ-binding motif
YAP	Yes Associated Protein
YIGSR	Tyrosine-isoleucine-glycine-serine-arginine
ZO-1	Zonula occludens-1
